# Biomechanical and biochemical evaluation of the effect of systemic application of omeprazole on the osseointegration of titanium implants

**DOI:** 10.1186/s40729-021-00310-5

**Published:** 2021-04-12

**Authors:** Samet Tekin, Serkan Dundar, Fatih Demirci, Alihan Bozoglan, Tuba Talo Yildirim, Nedim Gunes, Izzet Acikan, Erhan Cahit Ozcan

**Affiliations:** 1 Department of Prosthodontics, Faculty of Dentistry, Firat University, 23119 Elazig, Turkey; 2 Department of Periodontology, Faculty of Dentistry, Firat University, Elazig, Turkey; 3 Department of Prosthodontics, Faculty of Dentistry, Inonu University, Malatya, Turkey; 4 Department of Oral and Maxillofacial Surgery, Faculty of Dentistry, Dicle University, Diyarbakir, Turkey; 5 Department of Oral and Maxillofacial Surgery, Faculty of Dentistry, Sütcü Imam University, Kahramanmaras, Turkey; 6 Department of Plastic-Reconstructive & Esthetic Surgery, Faculty of Medicine, Firat University, Elazig, Turkey

**Keywords:** Osseointegration, Proton pump inhibitors, Omeprazole, Bone implant connection, Bone implant contact

## Abstract

**Background:**

This study aimed to investigate the effects of systemic omeprazole treatment on the osseointegration of titanium implants.

**Material and methods:**

After surgical insertion of titanium implants into the metaphyseal part of rats’ both right and left tibial bones, the animals were randomly divided into three equal groups: control (*n* = 8), omeprazole dosage-1 (*n* = 8) (OME-1), and omeprazole dosage-2 (*n* = 8) (OME-2) and totally 48 implants were surgically integrated. The rats in the control group received no treatment during the four-week postoperative experimental period. In the OME-1 and OME-2 groups, the rats received omeprazole in doses of 5 and 10 mg/kg, respectively, every 3 days for 4 weeks. After the experimental period, the rats were euthanized. One rat died in each group and the study was completed with seven rats in each group. Blood serum was collected for biochemical analysis, and the implants and surrounding bone tissue were used for biomechanical reverse-torque analysis. In the biomechanical analysis, implants that were not properly placed and were not osseointegrated were excluded from the evaluation.

**Results:**

One-way analysis of variance and Tukey’s honestly significant difference test and Student’s *t* test were used for statistical analysis. The reverse-torque test (control (*n* = 9), OME-1 (*N* = 7), and OME-2 (*n* = 7)) analysis of biochemical parameters (alkaline phosphatase, calcium, phosphorus, aspartate aminotransferase, alanine amino transferase, urea, and creatinine) revealed no significant differences between the groups (control (*n* = 7), OME-1 (*N* = 7), and OME-2 (*n* = 7)) (*P* > 0.05).

**Conclusions:**

Omeprazole had no biomechanical or biochemical effects on the osseointegration process of titanium implants.

## Background

Osseointegration is the direct structural and functional connection between an implant and a bone surface. The success of osseointegration depends not only on the surgical and prosthetic stage but also on the patient’s systemic condition. Early failure can occur even when conditions related to both the implant and the surgical protocol are favorable. Specific factors affecting the patient’s bone metabolism, such as age, gender, tobacco or alcohol use, systemic diseases, radiotherapy, and the use of certain systemic drugs, should be taken into consideration when evaluating the process of successful osseointegration, as they play an important role in early implant loss [1–3].

Pharmacological drugs taken systemically may have positive or negative effects on the osseointegration of dental implants [1–3]. Among them, proton pump inhibitors (PPIs) have been reported to impair the osseointegration of dental implants due to their negative effects on bone metabolism [1–5]. Such drugs, including omeprazole, lansoprazole, pantoprazole, rabeprazole, and esomeprazole, act by inhibiting the stomach’s proton pump function and suppressing gastric acid secretion. They are the most widely prescribed drugs worldwide for the treatment of reflux, esophagitis, peptic ulcers, dyspepsia, and other acid-related gastrointestinal diseases. They are also used to protect from adverse effects of non-steroidal anti-inflammatory drugs on the stomach [4–6]. However, as stomach acid and the mild acidic environment of the proximal duodenum are essential for calcium absorption from the duodenum, chronic suppression of stomach acid by proton pump inhibitors disturbs the balance of calcium and vitamin B12, leading to adverse consequences for the bones, such as bone loss, bone fractures, and poor bone quality [3–8].

Epidemiological studies have reported that long-term use and high doses of PPIs increase the risk of bone fractures [7, 8]. Reduced bone mineral content and density, cortical thickness, and biomechanical resistance of bones reported in various in vivo studies confirm the PPI–bone relation observed in clinical settings [7–11]. Additionally, some in vitro studies have reported that PPIs reduce bone resorption by inhibiting the vacuolar osteoclastic H+-ATPase enzyme [12, 13]. In contrast, it has also been reported that the long-term use of PPIs is not associated with changes in bone strength or mineral density [14].

Omeprazole is one of the most frequently prescribed PPIs orally. Due to its high first pass metabolism, the optimal efficacy and bioavailability of oral omeprazole is lower than that seen following intravenous administration. When omeprazole is taken orally, its bioavailability increases during the first 5 days of the treatment. The optimal drug efficacy and increase in bioavailability seen in omeprazole during this time is due to the reduction in first pass extraction. This is probably the result of decreased acid degradation in the stomach lumen due to increased gastric pH in the first few days of treatment and inhibition of the own metabolism of these drugs [6].

When the literature is examined, besides the studies reporting that omeprazole affects the bone tissue negatively, there are also studies stating that it affects positively [7–16]. Studies on the effects of omeprazole and other PPIs on dental implants are limited and continues to be investigated. Therefore, the aim of this study was to investigate the effect of systemic omeprazole treatment on bone–implant connections in rat tibia.

## Methods

### Animals and experimental design

All surgical and experimental procedures in this study were conducted at the Experimental Research Center of Firat University in Elazig, Turkiye. Approval for the study was granted by the university’s Animal Experimental Ethics Council (2019/146). The recommendations of the Declaration of Helsinki regarding the treatment of laboratory research animals were stringently followed.

For the purposes of this study, 24 healthy adult female Sprague-Dawley rats aged 2.5–3 months were used, selected from rats in the same estrus period after vaginal smear to ensure experimental protocol standardization. On the first day of the experimental period, their average body weight was 220–230 g. The rats were kept in plastic cages in conditions of 12-h dark/light cycles, and their temperatures were checked daily. Food and water were provided ad libitum throughout the experimental period. The number of animals were determined by power analysis in the experiments; 8% deviation, type 1 error (α) 0.05 and type 2 error (β) (power = 0.80), and if the animals were divided into groups, at least seven animals in each group should be determined. Eigth rats per group were included in the study due to the probability of some subjects dying during the surgical and experimental period.

### Surgical procedures and drug application

**Fig. 1:**
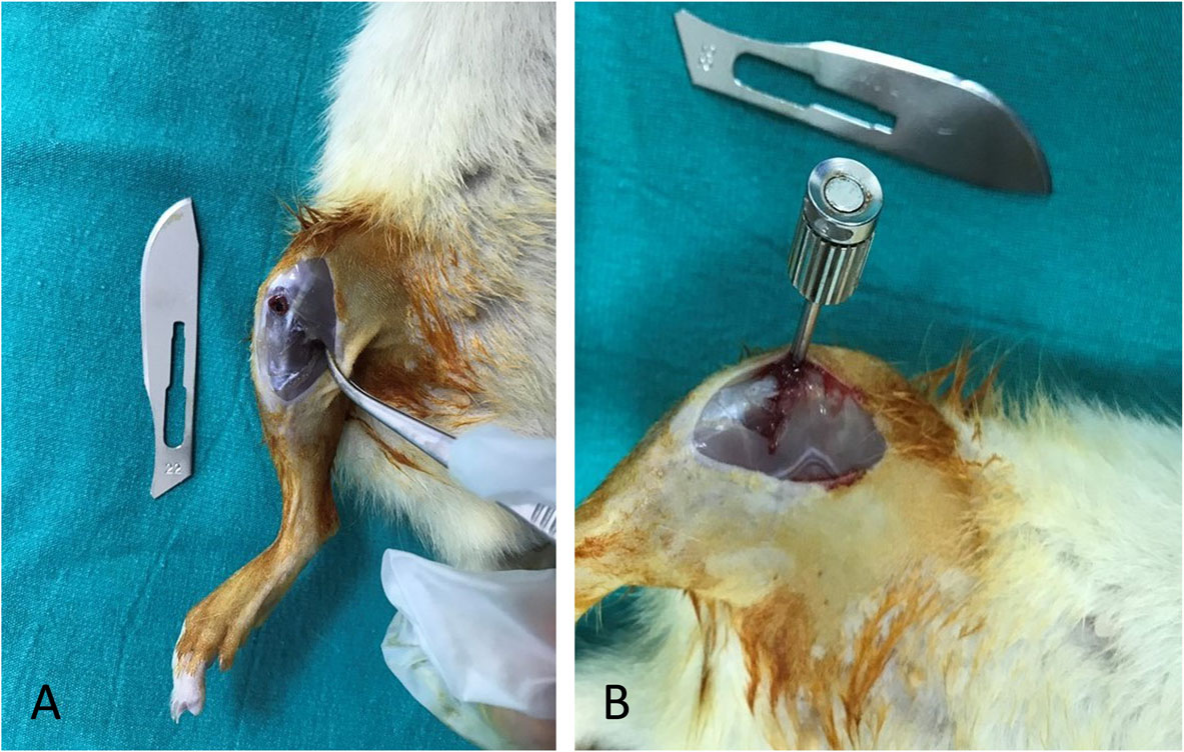
**a** Surgical preperation of the titanium implant sockets and **b** after insertion of the titanium implants in bone sockets

Titanium implants were surgically inserted into both right and left tibial bones of the rats. All surgical procedures were performed atraumatically by the same researcher under sterile conditions. Ketamine hydrochloride (40 mg/kg) and xylazine (10 mg/kg) were intramuscularly injected into the rats to induce general anesthesia. The surgical site was then washed with povidone-iodine and shaved. A 15-mm-long incision was made in the right and left tibial crests, and the soft tissue was dissected and incised to expose the tibial metaphyseal bone. Implant sockets were created using appropriate drills with saline perfusion. Totally 48 machined-surface titanium implants 4 mm in length and 2.5 mm in diameter were inserted into the metaphyseal part of the both right and left tibial bones in all groups, and primary stabilization was achieved [15]. Following the placement of the titanium implants, the flap was returned to the original position, and the fascia, subcutaneous tissue, and skin were sutured using 4-0 polyglactin sutures. To prevent pain and infection, an analgesic (0.1 mg/kg tramadol hydrocloride) and an antibiotic (50 mg/kg penicilin) were intramuscularly injected into each rat for three days postoperatively (Fig. 1a, b).

Upon completion of the surgical procedures, the rats were randomly divided into three groups with similar mean weights: a control (CNT) group (*N* = 8), an omeprazole dosage-1 (OME-1) group (*N* = 8), and an omeprazole dosage-2 (OME-2) group (*N* = 8). The rats in the CNT group received no treatment during the postoperative experimental period only saline application was done by orogastric catheter. In the OME-1 and OME-2 groups, omeprazole in doses of 5 and 10 mg/kg, respectively, was administered by oral gavage three times a week [15, 17].

Oral administration of omeprazole was made by means of an orogastric catheter and was given directly into the stomach of rats. Thus, all rats in the experimental groups were prevented from vomitting the given drug-omeprazole from their stomachs and a model similar to that of humans taking omeprazole orally was obtained. While the study continued, one rat died in each group and the study was completed with seven rats in each group.

### Biochemical analysis

Four weeks postoperatively, the animals were euthanized. Blood samples were collected from the rats under deep anesthesia by cardiac puncture without anticoagulant to measure the serum alkaline phosphatase (ALP), calcium (Ca), phosphorus (P), aspartate amino transferase (AST), alanine amino transferase (ALT), urea, and creatinine levels. The biochemical analysis was performed in the central biochemistry laboratory of the Faculty of Medicine of  Firat University.

### Biomechanical analysis

**Fig. 2:**
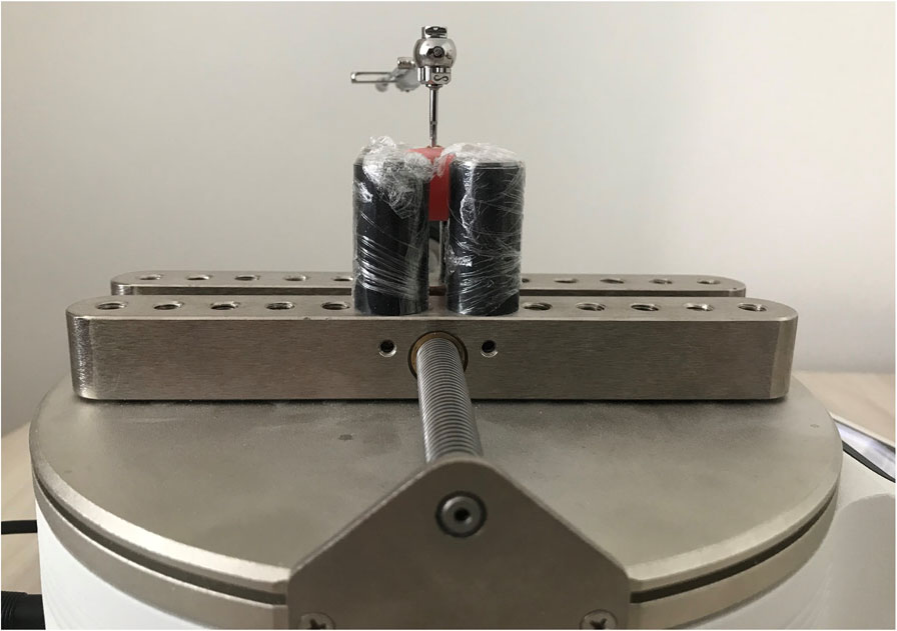
Reverse torque analysis of the titanium implants (Tonichi STC400CN, Buffalo Grove, IL, USA)

The titanium implants were removed from the soft tissue along with the surrounding bone tissue. The samples were prepared immediately after removal of the block bone fragment tibia containing the implants. They were placed in liquid solution (10% buffered formalin) and evaluated immediately to avoid dehydration. Then, the implants with the surrounding bone tissue were buried in polymethylmethacrylate. After a special screw piece was placed on the implants, a digital torque tool was fixed on each, and counterclockwise force was manually applied slowly and increasingly. The procedure was completed when the implant started to rotate inside the bone socket. The highest torque value (in Newton centimeters) obtained on the digital torque screen at the time of breaking was automatically recorded (Fig. 2).

### Statistically analysis

Statistical analysis was performed with SPSS Statistics 22.0 for Windows (IBM, Armonk, NY, USA). All values were expressed as means ± standard deviations. Differences between the groups were assessed using one-way analysis of variance (ANOVA). Tukey’s honestly significant difference (HSD) test was used to determine the group that caused these differences. Additionally Student’s *t* test was performed to analysis of the datas between controls and test animals. A value of *P* < 0.05 was considered statistically significant.

## Results

While performing biomechanical reverse-torque analysis, improperly placed and non osseointegrated implants were excluded from the study. Biomechanical analyzes were completed with nine implants in the control group and seven implants in the experimental groups. Although there are numerical differences between groups according to biomechanic reverse torque values, no statistically significant differences in the titanium implants were observed between the groups (Tables 1 and 2).

**Table 1:** Biochemical parameters of the groups

Parameters	Groups	N	Mean	Std. Deviation	Min.	Max	P*
ALP (U/L)	Control	7	55.86	21.47	32	88	0.098
	D_1	7	115.57	80.37	60	289	
	D_2	7	78.71	16.01	57	96	
Ca (mg/dl)	Control	7	7.92	2.17	5.26	10.36	0.09
	D_1	7	9.69	0.39	9.38	10.42	
	D_2	7	9.05	1.12	6.74	10.11	
P (mg/dl)	Control	7	5.51	1.43	3.70	6.80	0.221
	D_1	7	6.43	0.48	5.80	7.10	
	D_2	7	5.86	0.68	4.50	6.70	
AST (U/L)	Control	7	187.29	61.01	97	274	0.196
	D_1	7	256	95.76	171	413	
	D_2	7	211.86	36.73	146	252	
ALT (U/L)	Control	7	59.43	16.51	33	78	0.081
	D_1	7	89	36.85	61	166	
	D_2	7	65.14	12.51	43	79	
Urea (mg/dl)	Control	7	49	9.36	37.00	60	0.503
	D_1	7	53.57	6.45	44	63	
	D_2	7	51.43	4.96	42	57	
Creatinine (mg/dl)	Control	7	0.37	0.17	0.16	0.55	0.692
	D_1	7	0.50	0.07	0.43	0.64	
	D_2	7	0.45	0.12	0.23	0.59	

*One-way ANOVA. *ALP* alkaline phosphatase, *Ca* calcium, *P* phosphor, *AST* aspartate transaminase, *ALT* alanine transferase. *D_1* 5 mg/kg omeprazole, *D_2* 10 mg/kg omeprazole

**Table 2:** Biomechanic parameters of the groups

Parameter	Groups	*N*	Mean	Std. deviation	Min.	Max.	P*
Force (Ncm)	Control	9	0.8	1.1	0.1	3.10	0.692
	D_1	7	0.53	0.88	0.10	2.50	
	D_2	7	0.97	0.86	0.20	2.30	

*One-way ANOVA

Although there are numerical differences between groups according to biochemical datas, no statistical differences were detected between the groups in terms of AST, ALT, urea, creatinine, ALP, Ca, P levels, and biomechanic reverse torque values (Tables 3 and 4).

**Table 3:** Biochemical parameters of the groups

Parameters	Groups	*N*	Mean	Std. deviation	Std. error	P*
ALP (U/L)	Control	7	55.86	21.47	8.11	0.091
	(PPI D_1 + D_2)	14	97.14	58.87	15.73	
Ca (mg/dl)	Control	7	7.92	2.17	0.82	0.133
	(PPI D_1 + D_2)	14	9.37	0.87	0.23	
P (mg/dl)	Control	7	5.51	1.43	0.54	0.302
	(PPI D_1 + D_2)	14	6.14	0.64	0.17	
AST (U/L)	Control	7	187.29	61.01	23.06	0.164
	(PPI D_1 + D_2)	14	233.93	73.35	19.6	
ALT (U/L)	Control	7	59.43	16.51	6.24	0.157
	(PPI D_1 + D_2)	14	77.07	29.19	7.80	
Urea (mg/dl)	Control	7	49	9.36	3.54	0.389
	(PPI D_1 + D_2)	14	52.5	5.64	1.51	
Creatinine (mg/dl)	Control	7	0.37	0.17	0.07	0.17
	(PPI D_1 + D_2)	14	0.47	0.1	0.03	

*Student’s *t* test. *ALP* alkaline phosphatase, *Ca* calcium, *P* phosphor, *AST* aspartate transaminase, *ALT* alanine transferase, *PPI* proteon pump inhibitor_Omeprazole, *D_1* 5 mg/kg Omeprazole, *D_2* 10 mg/kg Omeprazole

**Table 4:** Comparisons of biomechanical parameters of the groups

Parameter	Groups	*N*	Mean	Std. deviation	P*^1,2,3^
Force (Ncm)	Control	9	0.80	1.1	> 0.05
	PPI D_1	7	0.53	0.88	
	PPI D_2	7	0.97	0.86	

*Student’s *t* test. *P*^1^ : 0.60 *(control–D_1), P*^2^ : 0.74 *(control–D_2)*, *P*^3^: 0.36 *(D_1–D_2)*

## Discussion

In this study, the effect of systemic administration of different omeprazole doses on the osseointegration of titanium implants on rat tibia was evaluated by both biochemical and biomechanical analyses. No effect was observed.

Al Subaie et al. used 24 Sprague-Dawley rats in their experimental study, a titanium implant was placed in the left tibia [15]. During the 2 weeks following surgery, 12 rats were treated with omeprazole (5 mg/kg, daily) and the other 12 with saline. They reported that omeprazole impaired the bone implant connection histologically. Due to this reason, we prefered 5 mg/kg and 10 mg/kg oral doses in experimental period according to Armah et al.’s and Al Subaie et al.’s research [15, 17].

In an animal study evaluating omeprazole’s osteogenic activity, Cottrell et al. [18] found no significant effect on bone formation. In another animal study, Hasanin et al. [11] reported that omeprazole had both negative and positive effects on bone remodeling, on the one hand reducing calcium absorption and bone formation, and on the other hand potentially inhibiting bone resorption by supressing the vacuolar osteoclastic H+-ATPase enzyme. Bodde et al. [16] reported that omeprazole treatment had no significant effect on bone formation. Joo et al. [10] evaluated the effects of long-term OME therapy on bone turnover in their in vivo study. They examined the signaling pathway involved in osteoclast differentiation and bone resorption/formation parameters experimentally in osteporosis model after the ovariectomy in female rats. Joo et al. [10] reported that there was no statistically significant difference in serum calcium levels among all groups. In general, it can be seen that serum calcium levels do not change substantially beyond the normal range in rats even though ovaries have been removed. As in our work, they thought that this result was similar to previous data showing that serum calcium can remain unchanged even under internal or external experimental conditions. Huang et al. [19] In their studies investigating the effects of calcitriol on bone mineral density in patients treated with esomeprazole, an isomer of omeprazole, they reported that serum ALP values did not make a statistical difference between the treatment and control groups. Our results-ALP are consistent with Huang et al's research. Serfaty-Lacrosniere et al. [20] investigated whether hypochlorhydria associated with treatment with omeprazole could affect intestinal absorption of calcium, phosphorus, magnesium, or zinc in their research in humans and they reported that no change in the intestinal absorption of phosphorus. But Al Subaie et al. reported that omeprazole-5 mg/kg with intraperitoneal injection daily negatively affected bone implant connection in a 2-week experimental protocol histologically [15]. But two different doses of omeprazole-5 mg/kg and 10 mg/kg with oral gavage, did not produce a significant effect on the osseointegration of titanium implants in our study.

Clinical studies have been conducted on the effect of PPIs on osseointegration. Altay et al. [4], retrospectively analyzing 1918 dental implants in 592 patients, found that the use of PPIs increased the risk of premature dental implant loss. Similarly, in a retrospective cohort study, Wu et al. [3] reported that PPIs increased the risk of failure of osseointegrated dental implants. In another retrospective cohort study involving 3559 dental implants and 999 patients, Chrcanovic et al. [21] reported that implant loss rates were 12% in PPI users and 4.5% in non-PPI users, concluding that PPI use had a negative effect. Aghaloo et al. [1] also found that PPIs had a negative effect on implant osseointegration. Mester et al. [5] reported a link between the use of PPIs and bone regeneration and osseointegration; however, factors such as recipient bone, surgical trauma, titanium surface limitations, comorbidities, and interaction with other pharmacological agents should be considered together. Although the overall effect of omeprazole on bone metabolism was similar in this study, only the tibial bone was evaluated. Bone healing and implant osseointegration are difficult to simulate in vitro, as they depend on cells, hormones, and systems. A rat tibia model was chosen for ease of application for implant integration; however, its structure is not similar to that of the mandible or the maxilla. Although the tibial bone is surrounded by thick and well-perfused muscles, it cannot fully imitate intraoral conditions. In addition, experimental rat model studies differ from human studies in terms of skeletal change and maturity and bone turnover and healing behavior [15, 16, 18, 22].

Reverse-torque analysis is used in animal and laboratory studies—but not clinically—to evaluate primary implant stability and osseointegration. Reverse-torquing the implant in the bone provides an indirect measurement of the force required to separate the bone–implant interface. It is an objective evaluation criterion for implants with different designs and surface features and for different bone healing conditions [23–25]. Reverse torque analysis is an application that allows the evaluation of the entire bone around the implant. Histological analysis, on the other hand, allows evaluation only on a specific and very thin section. In our study, the reverse-torque test was used to evaluate the effect of different postoperative doses of omeprazole on osseointegration according the literature [24, 25]. No difference was found between the doses.

Serum calcium and phosphorus levels are direct measures of the degree of mineralization during bone healing. In this study, there were no statistically significant differences in serum calcium and phosphorus levels between the control and the omeprazole groups. However, it should be remembered that bone density and mineralization degree alone are not sufficient for bone quality evaluation. Serum alkaline phosphatase is an important biochemical bone-building marker [10, 21]. Although the difference between the three groups was not statistically significant, a numerical increase in alkaline phosphatase levels was observed in the groups treated with omeprazole compared to the control group.

Although the biomechanical osseointegration levels and biochemical markers make a numerical difference between the groups, it is seen that there is no statistically significant difference between the groups. This result contradicts with the work of Al Subaie et al. [15]. In additon the histological examination can examining a specific cross section but biomechanic evaluation can evaluate the 3-dimensional response of the entire bone tissue around the implant. For this purpose, we preferred biomechanical analysis in our study.

This study has some limitations. First, the molecular mechanisms underlying the relationship between systemic omeprazole and osseointegration and bone tissue are not fully explained due to the method used in this study. Second, although experimental animal studies are necessary to explain the relationship between systemic omeprazole and bone tissue, the data from the results of these studies can only be used to predict the corresponding pathways in humans. Third, the survival rate or long-term success of implants could not be evaluated in this study. Fourth, in our skeletal system, long bones such as tibia-femur and craniofacial bones (mandible-maxilla) have different osteogenic potential and therefore may respond differently to systemic administration of omeprazole [26, 27].

## Conclusion

In this study, different doses (5 mg/kg and 10 mg/kg) of omeprazole had no effect on osseointegration. More studies are needed to assess the short- or long-term effects of omeprazole on bone healing and osseointegration. Data obtained from the evaluation of dental implants in patients using PPIs can be a guide for conducting further studies and developing new treatment protocols.

## Data Availability

The datasets used and/or analyzed during the current study are available from the corresponding author on reasonable request.

## Abbreviations

PPIs: Proton pump inhibitors
ALP: Alkaline phosphatase
Ca: Calcium
P: Phosphorus
AST: Aspartate amino transferase
ALT: Alanine amino transferase
ANOVA: One-way analysis of variance
HSD: Honestly significant difference
